# Characterisation of ocular involvement in an experimental model of neuroschistosomiasis mansoni

**DOI:** 10.1590/0074-02760190029

**Published:** 2019-07-15

**Authors:** Thiago Andre Alves Fidelis, Geraldo Brasileiro-Filho, Helena Hollanda Santos, Daniel Vitor Vasconcelos-Santos, Patricia M Parreiras, Paulo Marcos Z Coelho, Neusa Araujo, Marco Vinicius Chaud, José Roberto Lambertucci

**Affiliations:** 1 Universidade Federal de Minas Gerais, Faculdade de Medicina, Departamento de Doenças Infectoparasitárias, Belo Horizonte, MG, Brasil; 2 Universidade de Sorocaba, Laboratório de Biomateriais e Nanotecnologia, Sorocaba, SP, Brasil; 3 Universidade Federal de Minas Gerais, Faculdade de Medicina, Hospital das Clínicas, Departamento de Oftalmologia, Belo Horizonte, MG, Brasil; 4 Fundação Oswaldo Cruz, Instituto René Rachou, Laboratório de Esquistossomose, Belo Horizonte, MG, Brasil

**Keywords:** neuroschistosomiasis, ocular lesion, eye granuloma

## Abstract

The Global Burden of Disease Study 2010 listed schistosomiasis among the leading 100 causes of death in Brazil, responsible for 3.6% of the estimated total of deaths globally. Eye and adnexa are very rarely affected by schistosomiasis mansoni, with limited documentation of ocular pathology in this setting. This short communication reports ocular histolopathological findings in a murine model of neuroschistosomiasis mansoni. Lesions were found in the bulbar conjunctiva, lacrimal gland, choroid and corneoscleral limbus.

The World Health Organization estimates that between 200 and 300 million people worldwide are infected with *Schistosoma* spp and 800 million people in the world are at risk of infection. Approximately 280,000 deaths/year are attributed to schistosomiasis chronic complications.^1^ Ophthalmologic changes associated with schistosomiasis mansoni are rarely discussed in the literature. On fundus examination, Oréfice et al.^2^ identified bilateral lesions in the choroid and retina of five patients with hepatosplenic schistosomiasis. These were clinically characterised as multiple bilateral white-yellow nodules, apparently located in the choroidal plane. Moreover, the authors^2^ concomitantly provided the first histopathological documentation of such lesions, identified as choroidal granulomas containing *Schistosoma mansoni* eggs. Animal models may be instrumental to better understanding the complex pathogenesis of this fascinating disease.

We infected 25 male mice (*Mus musculus - Swiss Webster,* weighing between 18 and 20 grams) with 50 LE strain cercariae subcutaneously and 25 animals were maintained as controls (uninfected). All animals were followed for 160 days post-infection. At 88 (animal #1), 97 (animal #2) and 109 (animal #3) days post-infection, euthanasia procedures were performed (n = 2/group), by CO_2_ gas chamber, according to guidelines and principles of the Brazilian Council on Animal Care. The protocol was approved by the local Institutional Animal Care Committees at the Federal University of Minas Gerais and at the René Rachou Research Institute [Oswaldo Cruz Foundation (Fiocruz), state of Minas Gerais, Brazil]. The *ex vivo* samples had a catheter placed into the right heart and perfused by a fixative solution of 10% paraformaldehyde (PFA). Worm recovery was carried out as per the technique prescribed by Pellegrino and Siqueira.^3^ Experiments were performed on a 7T magnetic resonance scanner (MRI System 7T/210 ASR Horizontal Bore Magnet, Agilent Technologies, Palo Alto, CA, USA). *Ex vivo* brain images were obtained using 3D T1 Gre (TR/TE: 370 ms/5 ms, Matrix: 128 x 96 x 96, FA: 35º, Nex: 13, Fov: 20 × 15 × 15 mm, acquisition time: 12h 18min), coronal Multi Echo (TE/TR: 3,000/9 ms, 3 Echos, Nex: 30, Matrix: 128 × 128, Fov: 15 × 15 mm, Slices: 30, Slice Thickness: 0.5 mm, no Gap, acquisition time: 3h 12 min). After imaging, brain and skull were immersed in 7% nitric acid for decalcification. After one day (24 h), the whole skull was sectioned in 3 mm thick (frontal slices) and dove in 7% nitric acid for 24 h for complete decalcification. After that, the fragments were sectioned in 1.1 mm thick slices, each one placed in a paraffin block (10-11 blocks for each animal). Serial 4 µm sections (obtained from 50 µm intervals between each) from all paraffin blocks were stained with haematoxylin and eosin (H&E). Light microscope was used to search for any morphological lesion, especially *Schistosoma* eggs and/or granulomas. The right hemisphere of each animal’s skull was stained with Nankin^®^ ink for identification (Fig. 1). 

**Fig. 1:**
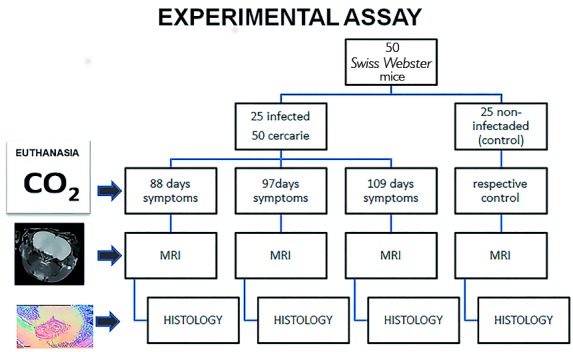
experimental essay of neuroschistosomiasis murine model. MRI: magnetic resonance scanner.

In 25 *Swiss Webster* mice subcutaneously infected with 50 cercariae of the *S. mansoni* (LE strain), two mice (animals #1 and #2) presented neurological manifestations such as spinning, hemiparesis and ataxia. Animal #3 remained without neurological signs (asymptomatic). Histology confirmed lesions in the brain associated with *S. mansoni* eggs in all three mice. Granuloma formation was noted, with infiltration of mononuclear (lymphocytes, plasma cells and macrophages), but also of polymorphonuclear (neutrophils) leukocytes. During histopathological study of the brain, we incidentally found eggs and granulomas in the bulbar conjunctiva, lacrimal gland, choroid and corneoscleral limbus (Fig. 2), successfully reproducing ocular/periocular infection of schistosomiasis mansoni in three of the 25 infected mice (12%). Prior magnetic resonance imaging (MRI) analysis had not identified these ocular/periocular changes. 

**Fig. 2A:**
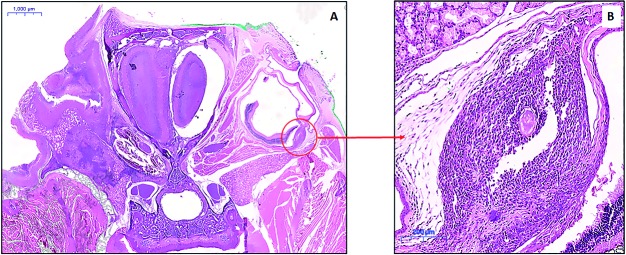
small increase (frontal cut) at the ocular bulb level haematoxylin and eosin (H&E); B: detail of the ocular bulb. Fusiform lesion is seen at the level of the choroid and sclera, medially to the optic nerve (H&E); granuloma formation is noted with *Schistosoma mansoni* egg in the centre (H&E). Inflammatory cells in the granuloma is not possible to be observed in Fig. 2B due to the small magnification of the image.

The model adopted in this study demonstrated granulomas in the encephalon and ocular/periocular region of infected mice, being the first characterisation of unequivocal ocular involvement in experimental model of neuroschistosomiasis mansoni. Most of the lesions are in the periocular topography, but the choroidoscleral granuloma (Fig. 2) is consistent with previous reports in humans.^2^ ^,^ ^4^ Interestingly, these could not be demonstrated on prior MRI scans, probably because of their small size. Remigio et al.^5^ have previously reported a presumed retinal granuloma in one out of 25 (4%) *Swiss* mice infected with *S. mansoni* (exposing the tails to a suspension of 40 cercariae). Ismail et al.^6^ found deposition of *S. mansoni* antigen in the eyes of 17 of 50 (35%) hamsters infected by *S. mansoni* cercarie. Their results detected antigen in the retina, lacrimal gland and at subepithelial lining of the conjunctival sac. Marked subchoroidal and scleral antigen deposition and immune complexes were also revealed, even in the absence of detectable *Schistosoma* eggs in all of those regions. Constitutional melanin distribution at many of those sites, however, made it difficult to differentiate it from immunoperoxidase staining indicating *S. mansoni* antigens.

To the best of our knowledge, this is the first characterisation of unequivocal ocular involvement in experimental murine schistosomiasis. Further studies with this experimental model may help shed light to pathophysiology of ocular changes associated with this fascinating disease.

## References

[1] Fidelis TAA, Parreiras P, Tovar-Moll F, Meireles F, Brasileiro G, Coelho PMZ (2018). Murine model of neuroschistosomiasis mansoni: clinical,
histological and magnetic resonance imaging studies. Adv Tech Biol Med.

[2] Oréfice F, Simal CJ, Pittella JE (1985). Schistosomotic choroiditis I. Funduscopic changes and
differential diagnosis. Br J Ophthalmol.

[3] Pellegrino J, Siqueira AF (1956). A perfusion techinic for recovery of Schistosoma mansoni from
experimentally infected guinea pigs. Rev Bras Malariol Doencas Trop.

[4] Pittella JE, Oréfice F (1985). Schistosomotic choroiditis II. Report of first
case. Br J Ophthalmol.

[5] Remígio MCA, Brandt CT, Pontes-Filho NT, Albuquerque MCPA (2009). Histological and histomorphometric evaluation of the retina of
mice infected with Schistosoma mansoni in its hepatosplenic
form. Acta Bras Cir.

[6] Ismail HI, Ashour DS, Abou Rayia DM, Ali AL (2016). Ocular pathological changes in hamsters experimentally infected
with Schistosoma mansoni. J Helminthol.

